# Supplementary material for: Detoxification and Excretion of Trichothecenes in Transgenic Arabidopsis thaliana Expressing Fusarium graminearum Trichothecene 3-O-acetyltransferase
Source: Toxins (Basel). 2021 Apr 29;13(5):320. doi: 10.3390/toxins13050320 (PMC8145220; doi:10.3390/toxins13050320)
Supplement: Supplementary file 1 [file toxins-13-00320-s001.zip › toxins-1212369-supplementary.pdf]

toxins

MDPI

Supplementary Detoxification and excretion of trichothecenes in transgenic *Arabidopsis thaliana* expressing *Fusarium graminearum* trichothecene 3-O-acetyltransferase

Table S1. The copy number of *FgTRI101* in transgenic *Arabidopsis*.

Line	Ratio ±SD	Estimated *FgTRI101* copy number
FgTri101-2-T0	0.71±0.02	1
FgTri101-5-T0	0.68±0.01	1
FgTri101-6-T0	1.15±0.03	2
FgTri101-8-T0	0.63±0.02	1
FgTri101-9-T0	0.62±0.02	1
FgTri101-10-T0	0.70±0.03	1
FgTri101-12-T0	0.64±0.01	1
FgTri101-17-T0	0.80±0.08	1
FgTri101-19-T0	0.75±0.00	1
FgTri101-8-T3	0.96±0.00	1
FgTri101-12-T3	0.89±0.02	1
FgTri101-17-T3	1.05±0.02	1

Table S2. Primers used in the study.

Gene name	Primer sequence
**For transgenic plants**	
FgTRI101-ORF5'	5'-AGTCCCGGGCCACCATGGCTTTTCAAGATACAGCTC-3'
FgTRI101-ORF3'	5'-GAGACTAGTCTAACCGTACTGCGCATA-3'
D35S-For	5′-GACGCACAATCCCACTATCC-3′
Nos-Rev	5′-TTTGCGCGCTATATTTTGTTT-3'
4HPPD-For	5′-TGAGAGAGATGAGGAAGAGGAG-3′
4HPPD-Rev	5′-CCGACCCGTTTCTTGAGATT-3′
**For gene expression**	
FgTRI101-RT-F	5′-AAGGTGGGACTCTGGGATTA-3′
FgTRI101-RT-R	5′-GCTCTCAACAGGCTCAAAGA-3′
EF1α-RT-F	5′-TCTTGCCTGCTTTGAATGGA-3′
EF1α-RT-R	5′-TGTGAGGTCAACCACTGCGACAT-3′

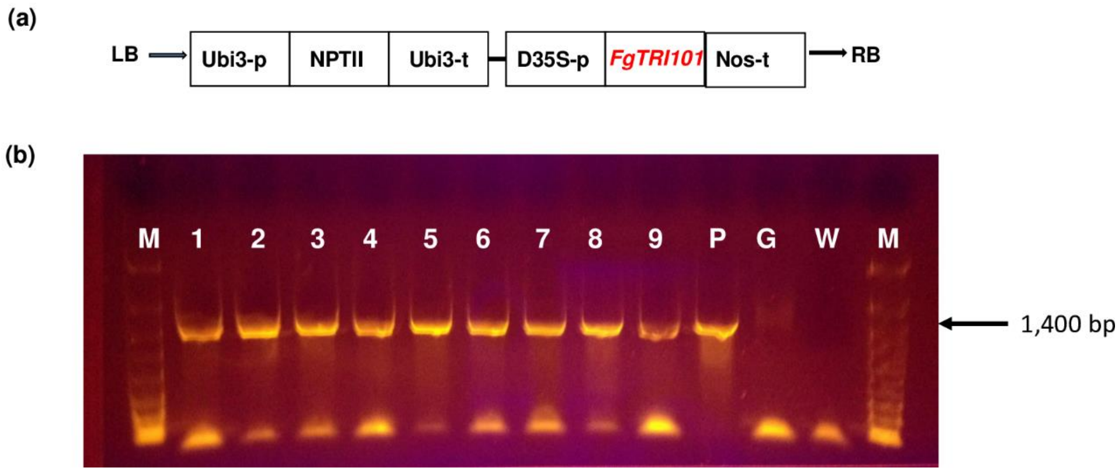

Figure S1. (a) Diagram of *FgTRI101* construct in the binary vector pBinARS/PLUS. Designations: LB, left border; Ubi3-p, promoter for NPTII; NPTII, neomycin phosphotransferase gene conferring kanamycin resistance; Ubi3-t, terminator for NPTII; D35S-p, double CaMV 35S promoter for *FgTRI101*; Nos-t, Nos terminator for *FgTRI101*. RB, right border. **(b)** PCR amplification of *FgTRI101* gene from transgenic *Arabidopsis* plants. Genomic DNA was amplified with *FgTRI101* primers ORF5′ and ORF3′. M: DNA marker; P: Positive control using plasmid as template; G: GUS-transformed plant as negative control; W: Water; Lane 1-9: Transgenic plants containing *FgTRI101*. Arrow indicates the size of PCR product.

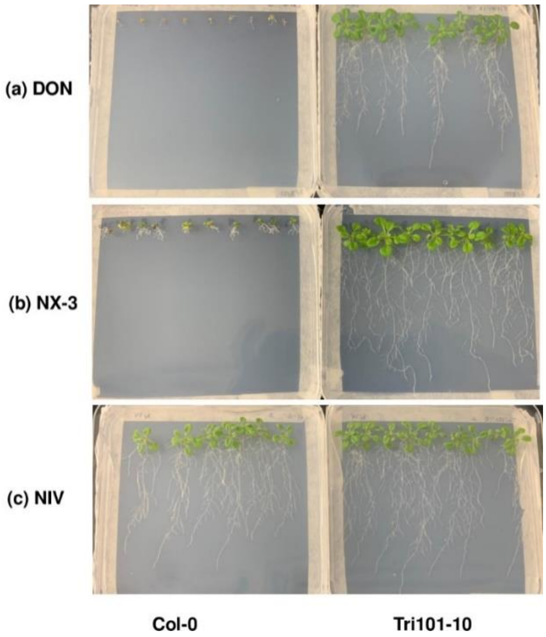

Figure S2. *Arabidopsis* seedling growth on MS media containing different trichothecenes (10 mg/L). (**a**) DON; (**b**) NX-3; and (**c**) NIV. Transgenic lines FgTri101-10 and the wildtype *Arabidopsis* Col-0 were used. The photographs were taken after a two-week incubation.

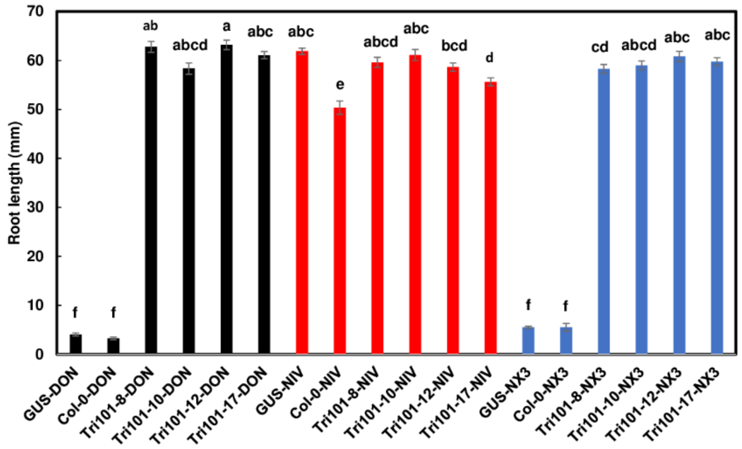

Figure S3. Root length comparison after a two-week incubation on MS media containing toxins. *Arabidopsis* Col-0, transgenic lines GUS, Tri101-8, 10, 12 and 17 were grown in MS media containing 10 mg/L DON, NX-3 or NIV respectively. The data were analyzed by one-way ANOVA and Tukey's post-hoc test using JMP. Bars with different letters indicate statistically significant differences.

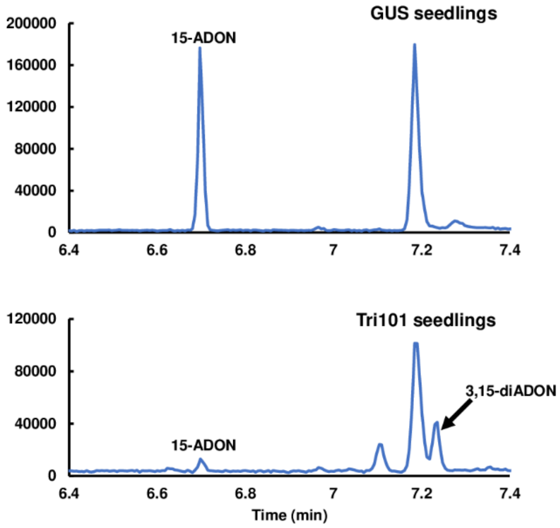

Figure S4. Chromatographs show 15-ADON converted to 3,15-ADON by FgTri101 transgenic. Scheme 50. mg/L 15-ADON in half MS liquid media for 2 days.

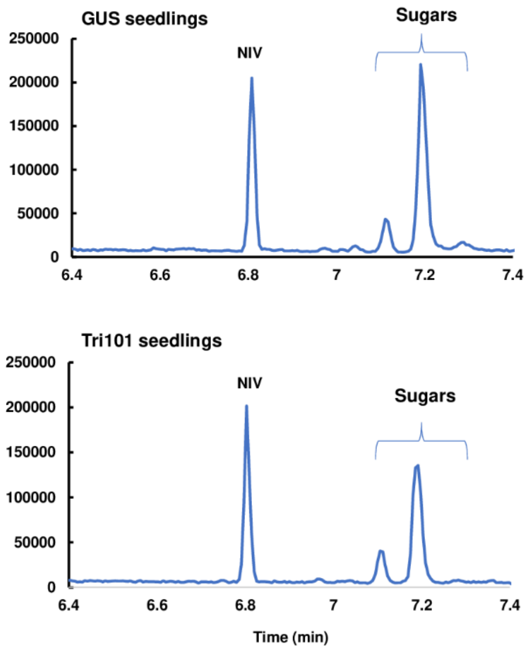

Figure S5. Chromatographs show no detectable acetylated NIV by FgTri101 transgenic *Arabidopsis*. *Arabidopsis* seedlings were treated with 50 mg/L NIV in half MS liquid media for 2 days. Peaks labeled as sugars were tentatively identified (e.g. sucrose, glucose, fructose) by comparisons with a NIST11 mass spectral library.

```
                     10        20        30        40        50        60
            ....|....|....|....|....|....|....|....|....|....|....|....|
F.graPH1    MAFKIQLDTLGQLPGLLSIYTQISLLYPVSDSSQYPTIVSTFEQGLKRFSEAVPWVAGQV
F.gra29149  ...............................P............................
F.gra38371  ............................................................
F.aca34207  ...............................P............................
F.asi13818  ...............................P............................
F.aus28285  ...............................P....A.......................
F.cer25805  ...............................P............................
F.cor29297  ...............................P............................
F.dac29298  .V...E.........................P.........L..........F.......
F.ger36905  ...............................P............................
F.ger38380  ...............................P............................
F.ger38405  ...............................P............................
F.lou54196  ...............................P............................
F.lou54197  ...............................P............................
F.lun13393  ...............................P............................
F.mer28436  ...............................P............................

                     70        80        90       100       110       120
            ....|....|....|....|....|....|....|....|....|....|....|....|
F.graPH1    KAEGISEGNTGTSFIVPFEDVPRVVVKDLRDDPSAPTIEGMRKAGYPMAMFDENIIAPRK
F.gra29149  ............................................................
F.gra38371  ............................................................
F.aca34207  ...........................................T................
F.asi13818  ............................................................
F.aus28285  ............................................................
F.cer25805  ............................................................
F.cor29297  ............................................E...............
F.dac29298  ...............I.......L.A..................................
F.ger36905  ............................................................
F.ger38380  ............................................................
F.ger38405  ............................................................
F.lou54196  ............................................................
F.lou54197  ............................................................
F.lun13393  ............................................................
F.mer28436  ............................................................

                    130       140       150       160       170       180
            ....|....|....|....|....|....|....|....|....|....|....|....|
F.graPH1    TLPIGPGTGPDDPKPVILLQLNFIKGGLILTVNGQHGAMDMVGQDAVIRLLSKACRNDPF
```

```
F.gra29149  ............................................................
F.gra38371  ..................................H.........................
F.aca34207  ..................................H.........................
F.asi13818  ............................................................
F.aus28285  ..................................H.........................
F.cer25805  ..........N.......................H.........................
F.cor29297  ..................................H.........................
F.dac29298  ..........N...................................M.............
F.ger36905  ..................................H.........................
F.ger38380  ..................................H.........................
F.ger38405  ..................................H.........................
F.lou54196  ..................................H.........................
F.lou54197  ..................................H.........................
F.lun13393  ..........N.......................H.........................
F.mer28436  ..................................H.........................

                     190       200       210       220       230       240
            ....|....|....|....|....|....|....|....|....|....|....|....|
F.graPH1    TEEEMTAMNLDRKTIVPYLENYTIGPEVDHQIVKADVAGGDAVLTPVSASWAFFTFSPKA
F.gra29149  ..................................P...................K.....
F.gra38371  ..................................P.........................
F.aca34207  ..................................P.........................
F.asi13818  ..................................P...................K.....
F.aus28285  ..................................P.........................
F.cer25805  ....K.............................P.........................
F.cor29297  ..................................P.........................
F.dac29298  ....KS........V..F....K.........I.P.........................
F.ger36905  ..................................P.........................
F.ger38380  ..................................P.........................
F.ger38405  ..................................P.........................
F.lou54196  ..................................P.........................
F.lou54197  ..................................P.........................
F.lun13393  ....K...S.........................P.........................
F.mer28436  ..................................P.........................

                     250       260       270       280       290       300
            ....|....|....|....|....|....|....|....|....|....|....|....|
F.graPH1    MSELKDAATKTLDASTKFVSTDDALSAFIWKSASRVRLERIDGSAPTEFCRAVDARPAMG
F.gra29149  ............................................................
F.gra38371  ............................................................
F.aca34207  ............................................................
F.asi13818  ............................................................
```

```
F.aus28285   ............................................................
F.cer25805   ............................................................
F.cor29297   ............................................................
F.dac29298   .............P.......................F...................P..
F.ger36905   ............................................................
F.ger38380   ............................................................
F.ger38405   ............................................................
F.lou54196   ............................................................
F.lou54197   ............................................................
F.lun13393   ........................................M...................
F.mer28436   ............................................................

                   310       320       330       340       350       360
             ....|....|....|....|....|....|....|....|....|....|....|....|
F.graPH1     VSNNYPGLLQNMTYHNSTIGEIANESLGATASRLRSELDPASMRQRTRGLATYLHNNPDK
F.gra29149   ............................................................
F.gra38371   ............................................................
F.aca34207   ............................................................
F.asi13818   ............................................................
F.aus28285   ............................................................
F.cer25805   ..................V.........................................
F.cor29297   ............................................................
F.dac29298   ..................V......P...............R..................
F.ger36905   ............................................................
F.ger38380   ............................................................
F.ger38405   ............................................................
F.lou54196   ............................................................
F.lou54197   ............................................................
F.lun13393   ..................V.........................................
F.mer28436   ............................................................

                   370       380       390       400       410       420
             ....|....|....|....|....|....|....|....|....|....|....|....|
F.graPH1     SNVSLTADADPSTSVMLSSWAKVGLWDYDFGLGLGKPETVRRPIFEPVESLMYFMPKKPD
F.gra29149   ...............................F............................
F.gra38371   ...............................F............................
F.aca34207   ...............................F............................
F.asi13818   ...............................F............................
F.aus28285   ...............................F............................
F.cer25805   ...............................F............................
F.cor29297   ...............................F............................
F.dac29298   ..............I.........C.E....F...........R................
```

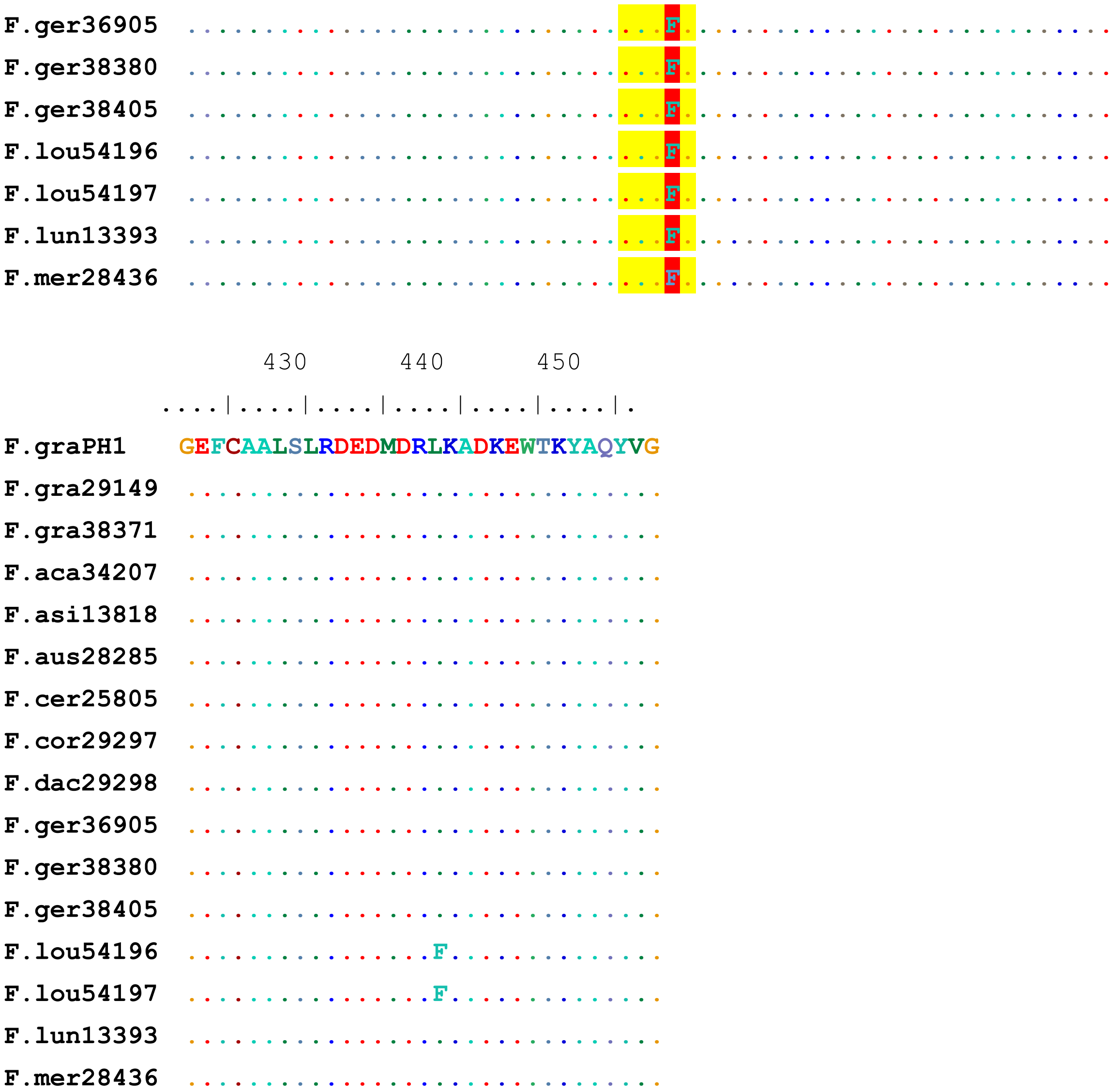

Figure S6. Comparison of deduced Tri101 amino acid sequences from DON producing strain PH-1 and NIV producing strains from *Fusarium* family. The sequences were aligned by BioEdit Sequence Alignment Editor program. The different amino acids between DON and NIV strains are shaded in red. The predicted catalytic sites and the motif DFGWG predicted for are shaded in yellow. All Tri101 sequences from NIV strains were extracted from whole-genome sequences of *Fusarium* isolates.
